# ORAL HEALTH PROMOTION IN PATIENTS WITH MORBID OBESITY AFTER GASTROPLASTY: A RANDOMIZED CLINICAL TRIAL

**DOI:** 10.1590/0102-672020190001e1437

**Published:** 2019-08-26

**Authors:** Ilma Carla de Souza PORCELLI, Nathalia Maciel CORSI, Marina de Lourdes Calvo FRACASSO, Renata Corrêa PASCOTTO, Alexandrina Aparecida Maciel CARDELLI, Regina Célia POLI-FREDERICO, Daoud NASSER, Sandra Mara MACIEL

**Affiliations:** 1 Postgraduate Program in Integrated Dentistry, State University of Maringá (UEM), Maringá, PR;; 2 Postgraduate Program in Communication, State University of Londrina (UEL), Londrina, PR;; 3 Program of Graduate Nursing, State University of Londrina (UEL), Londrina, PR;; 4 Department of Dentistry Program, University of Northern Paraná (UNOPAR), Londrina, PR, Brazil.

**Keywords:** Oral health, Health promotion, Health education, Bariatric surgery, Saúde bucal, Promoção da saúde, Educação em saúde, Cirurgia bariátrica

## Abstract

***Background*:**

The bariatric surgery may have negative repercussions on oral conditions.

***Aim*:**

To evaluate the impact of oral health educational/preventive program developed with patients submitted to gastroplasties.

***Method*:**

The sample consisted of 109 patients randomly allocated to two groups: intervention group (IG), where they participated in the oral health promotion program that include multiple educational-preventive approaches; control group (CG), where they received usual care from the bariatric clinic staff, without participation in the program. The oral conditions investigated in the pre-operative and postoperative periods of one month (1M) and six months (6M) were: dental caries, periodontal disease, tooth wear, dental plaque and salivary flow.

***Results*:**

After bariatric surgery, patients in IG presented: fewer changes in enamel (6M: p=0.004), dentin (6M: p=0.005) and gingival bleeding (6M: p<0.0001), reduction in plaque index (1M, 6M: p<0.0001) and increased salivary flow (6M: p=0.039), when compared with CG. Incipient tooth wear was recorded in both groups (6M: p=0.713).

***Conclusion*:**

There was a positive impact of the implemented program in the prevention of the main oral health problems in patients who underwent gastroplasties, contributing to their quality of life.

## RESUMO

***Racional*:**

A cirurgia bariátrica pode repercutir negativamente nas condições bucais.

***Objetivo*:**

Avaliar o impacto de um programa educativo/preventivo em saúde bucal desenvolvido em pacientes submetidos à gastroplastia.

***Método*:**

A amostra foi constituída por 109 pacientes aleatoriamente alocados em dois grupos: grupo de intervenção (GI), onde participaram do programa de promoção de saúde bucal com abordagens educativo-preventivas; grupo controle (GC), onde receberam cuidado da equipe da clínica, sem participação no programa. As condições bucais investigadas nos períodos pré e pós-operatório de um mês (1M) e seis meses (6M) foram: cárie dentária, doença periodontal, desgaste dentário, placa dentária e fluxo salivar.

***Resultados*:**

Após a gastroplastia, pacientes do GI apresentaram: menor alteração em esmalte (6M: p=0,004), dentina (6M: p=0,005) e sangramento gengival (6M: p<0,0001); redução no índice de placa (1M, 6M: p<0,0001) e aumento do fluxo salivar (6M: p=0,039), quando comparados aos do GC. Desgaste dentário incipiente foi registrado em ambos os grupos (6M: p=0,713).

***Conclusão*:**

Houve impacto positivo do programa implementado na prevenção dos principais problemas de saúde bucal em pacientes submetidos à gastroplastia, contribuindo para sua qualidade de vida.

## INTRODUCTION

In recent decades, obesity (body mass index BMI=30 kg/m²) rates have dramatically increased worldwide, in such a way that the World Health Organization has classified this trend as a real obesity “epidemic”^3^. Substantial evidence exists demonstrating that obesity is associated with and represent risk factor for a number of chronic diseases across the life cycle^8^. Morbid obesity (BMI=40kg/m²) entails far more serious health consequences for patients and creates additional challenges for healthcare providers^26^.

Patients with morbid obesity are at increased risk of mortality compared to non-obese, which explains the significant increase in the indication of bariatric surgeries^15^. According to the Brazilian Bariatric and Metabolic Surgery Society, 105.642 surgeries were performed in the private sector in 2017, an increase of 5.6% in relation to 2016. In the same period, the increase was 13.5% in the public sector^25^. These numbers set the country in second place in the world ranking, behind only the United States^2^.

 Surgery for the treatment of morbid obesity results in greater improvement in weight loss outcomes and reflects positively on treatment or control of comorbidities that are usually associated with it, such as diabetes, sleep apnea, arterial hypertension, dyslipidemia, coronary diseases, cancer and osteoarthritis, among others^15^. Moreover, there is a significant improvement in self-esteem in patients who have undergone gastroplasty^14^. Nevertheless, there are negative consequences of it, such as nutritional deficiencies_,_ the “dumping” syndrome (nausea, vomiting, redness, epigastric pain, hypoglycemia symptoms by the ingestion of simple carbohydrates), and eating disorders, such as anorexia, bulimia and compulsive eating, situations that have a direct influence on the oral cavity^14^ ^,^ ^15^.

 The literature has correlated gastroplasty with various oral problems, such as periodontal disease^21^ increase in dental caries^9^, hyposalivation, perimolysis, aphtha, dentin sensitivity, halitosis and alveolar bone loss as a consequence of osteoporosis^5^ ^,^ ^18^.

 Scarce longitudinal studies in the medical and dental literature have related the impact of the complications of bariatric surgery on oral health^18^, justifying new researches that may bring to light relevant data for promoting the oral health of these patients. Furthermore, the literature lacks studies on the effect of specific strategies, such as the elaboration of care protocols with educational-preventive guidelines used to prevent the most frequent oral problems in patients that have undergone gastroplasty.

 Considering that the determinants of oral diseases are known - which are common risk factors for other chronic diseases - such as diet, lack of hygiene, smoking, alcohol consumption, risk behaviors causing injuries and stress^23^, oral health should be integrated into the strategies of general health promotion, thereby providing gains in the quality of life and well-being of gastroplasty patients.

 In this context, this study was developed with the purpose of elaborating, implementing and evaluating a health promotion strategy to improve the oral health conditions of patients who had undergone gastroplasty.

## METHOD

This study was a randomized, controlled clinical trial following a parallel design. It was registered in the Brazilian Clinical Trial Register (“Registro Brasileiro de Ensaios Clínicos” - *ReBEC) under Number: RBR-2KCH38,* after having received approval by the Research Ethics Committee Process number 1.113.842.

The study population comprised obese individuals of both genders, who had been referred for having bariatric surgery performed between the months of February and July 2016, in three Morbid Obesity Surgery Centers, two located in Maringá, PR, and one in Campo Mourão, PR, both municipalities of southern Brazil. For surgical indication, all of them met one of the following conditions: having a BMI greater than 40 kg/m², above 35 kg/m² with comorbidities, or between 30 and 35 kg/m², in the presence of comorbidities classified as “severe” by the specialist phyisician^25^. Patients attend theses centers approximately two weeks prior to their surgery for a medical evaluation, at which time they were recruited to participate in the research.

A total of 160 patients had scheduled bariatric surgery for the period defined in the study and were evaluated for eligibility to participate in the research. In addition to having the surgery conducted in the established period, inclusion criteria were age between 16-60 years, signing of the informed consent form and availability to attend the consultations performed throughout the study. The main exclusion criteria were presence of edentulism, difficulties in seeing and hearing, illiteracy and physical and/or mental limitations.

Patients who fulfilled the inclusion criteria and agreed to participate in the research comprised the study sample (n=109), characterized as being of convenience. By means of the opening of sealed opaque envelopes that contained inside the acronyms IG and CG, provided by two trained nurses before the oral health evaluations, the participants were randomly allocated to the intervention group (IG), where they participated in an oral health promotion program (n=55), or to the control group (CG), where they received the usual care from the bariatric clinic staff, without participation in the program (n=54).

The assessment of the oral health conditions was carried out by a single research author (blinded) in the preoperative (baseline) and postoperative period of one month (1M) and six months (6M) after bariatric surgery. Clinical examinations were performed with both the examiner and person being examined seated, under light-focused illumination, with the use of an air syringe, a flat oral mirror, World Health Organization Probe (CPI probe)^28^ and gauze. The records were taken by a single annotator on individualized form.

The oral conditions evaluated and respective indices used were: dental caries (*International Caries Detection and Assessment System*)^11^, periodontal disease (*Community Periodontal Index*)^28^, tooth wear (*Tooth Wear Index*) adapted by Sales-Peres et al.^22^, dental plaque (*O’Leary’s Plaque Score Index)* ^20^ and salivary flow^7^.

By means of anthropometric measurements (weight and height), the body mass index (BMI) was calculated at baseline and 6M after bariatric surgery, and the patients were classified, observing the cut-off points recommended by the World Health Organization^30^: underweight (BMI<18.5 kg/m^2^), normal weight (18.5=BMI<25 kg/m^2^), pre-obese (25=BMI<30 kg/m^2^), obese (BMI=30 kg/m^2^) and morbidly obese (BMI=40 kg/m^2^). The surgical technique used in gastroplasty *(Roux-en-Y gastric bypass -* BGRY *or gastric sleeve*) was also recorded in the individualized form.

The sociodemographic information was obtained through personal interviews with participants, conducted at baseline, using a structured questionnaire that comprised the following variables: gender, age, marital status, education level, occupational condition and economic class (A1, A2, B1, B2, C1, C2, D and E), which were based on the Brazil Economic Classification criterion^1^. For statistical purposes, classes were grouped into the following categories: “upper” (A1, A2 and B1), “middle” (B2) and “lower” (C1, C2). No patient was classified in classes D or E.

After the oral evaluation, in order to stimulate participation in the research, a preventive kit from Curaprox - Swiss Dental (toothbrush CS 5460, Prime Plus interdental brush and Enzycal dentifrice), was distributed to all members of the IG and CG, in the preoperative consultation.

The educative-preventive interventions were performed by three other previously trained research authors. According to the protocol established in the oral health promotion program, at the preoperative consultation, they distributed to the participants of the IG a printed leaflet containing basic care guidelines for oral health maintenance, which were explained in detail and in an individualized way.

In the postoperative period, the educational-preventive interventions continued, directed only towards patients in IG. Individualized educational actions were developed during the consultations for the bariatric surgery revision, scheduled after one and three months. In addition, guidelines on oral health care were reinforced on a monthly basis by telephone contacts until the six months of follow-up were completed^10^. The telephone script was drawn by the research team. It contained clarification of doubts, questions about difficulties encountered and motivation to carry out the self-care on oral health proposed in the educational approach.

To ensure that the educational actions, and the preventive practices were up to date and correct, they were based on current scientific evidence^6^. For the prevention of dental caries, periodontal diseases and tooth erosion, the authors tried to pass simple and clear messages to the patients about the following topics: ingestion of a healthy, balanced diet (reduction in the quantity and frequency of foods and beverages with added sugar, avoid eating at night); adequate oral hygiene to remove bacterial plaque (brushing all the tooth surfaces and gingival margin carefully, with gentle movements, at least twice a day, with one of these times being before going to sleep; use a small amount of tooth paste, opt for fluoridated tooth paste; choose a tooth brush with a small head and medium bristles, which must be changed every three months; use dental floss to clean the interdental spaces; stimulate salivary flow to avoid dry mouth (increase water ingestion by taking a bottle with you and drinking small sips; increase the consumption of foods rich in fiber; chew gum without sugar, but only two month after surgery; if necessary, make use of artificial saliva; to avoid halitosis or coated tongue, brush the tongue or use a tongue scraper); take care to avoid tooth wear (diminish consumption of acidic foods, such as citrus fruit, vinegar and soft drinks (sodas); drink soft drinks or fruit juices through a straw, to minimize contact with the teeth; in case of ingesting soft drinks, never brush right afterwards, but perform mouth rinsing with water; never brush the teeth after episodes of vomiting or reflux, if you are not at home, perform mouth rinsing with water or chew gum without sugar; if you are at home: perform mouth rinsing with sodium bicarbonate (one teaspoon in half a glass of water, to alkalinize the oral medium, and wait for half an hour before brushing your teeth).

The professional preventive actions included supervised oral hygiene for plaque control, with the purpose of preventing caries and periodontal disease, which was performed in the post-operative periods of one (1M) and three months (3M) and topical varnish application with 5% fluoride Clinpro White Varnish (3M ESPE) on the teeth (postoperative of 1M). Both actions were realized at the morbid obesity surgery centers. It is worth mentioning that, at the 3M postoperative visit, a new preventive kit from Curaprox was distributed only to GI participants.

### Statistical analysis

Data were processed and analyzed by the Statistical Package for Social Science (SPSS), version 20.0 software was used. By univariate analysis, the possible associations between the study variables were verified. For comparisons between CG and IG the Mann-Whitney, Chi-square and Exact Fisher tests were used. The significance level was set at 5%. The diagnostic criteria used in the dental caries evaluation were recoded according to the localization of the lesions into: without caries (ICDAS=0), caries in enamel (ICDAS=1 and =3); and caries in dentin (ICDAS=4 and =6). The author who performed the statistical procedures was blinded during the analysis. Only after data collection was complete one of the researcher broke the randomization code to input the group allocation within the pre-existing data set.

## RESULTS

**FIGURE 1:**
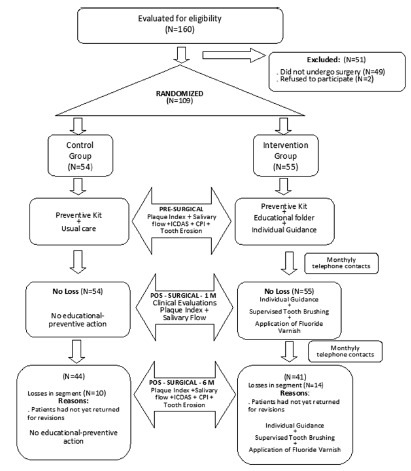
Flow chart of the trial (CONSORT statement)

Of the total number of bariatric patients who began the research (n=109), 78% underwent the third evaluation in the postoperative period of six months (n=85). Figure 1 shows the number of participants in each stage of the research, and the procedures performed in each stage.

At the time of baseline assessment, the mean age of the participants in the intervention group (IG) was 36.5±10.9 and in the control group (CG), 33.4±9.5 years. In Table 1, it may be observed that the groups IG and CG were homogeneous relative to demographic and socioeconomic characteristics and type of surgery of the participants (p>0.05). In both groups, the authors noted a higher proportion of females, who had partners, with high educational level, remunerated occupation and who underwent the surgical bypass technique. In addition, similar proportions of participants in the three social classes were observed.

**TABLE 1:** Distribution of bariatric patients according to sociodemographic variables and type of surgery performed (n=109)

Indicators	Groups		
	Control	Intervention	p
	n (%)	n (%)	
Gender			0.294*
Male	18 (33.3)	13 (23.6)	
Female	36 (66.7)	42 (76.4)	
Age			0.068**
17 to 27 years	16 (29.6)	12 (21.8)	
28 to 34 years	20 (37.0)	11 (20.0)	
35 to 44 years	10 (18.5)	17 (30.9)	
45 years or older	8 (14.8)	15 (27.3)	
Marital status			0.426
With partner	33 (61.1)	38 (69.1)	
Without partner	21 (38.9)	17 (30.9)	
Educational level			0.110**
5 to 8 years	9 (16.7)	4 (7.3)	
9 to 11 years	20 (37.0)	19 (34.5)	
12 years or more	25 (46.3)	32 (58.2)	
Occupational condition			0.818*
Remunerated	43 (79.6)	42 (76.4)	
Not remunerated	11 (20.4)	13 (23.6)	
Economic class			0.665**
Upper	12 (25.0)	16 (32.0)	
Middle	17 (35.4)	18 (36.0)	
Lower	19 (39.6)	16 (32.0)	
Type of surgery			0.665*
Sleeve	13 (24.1)	16 (29.1)	
Bypass	41 (75.9)	39 (70.9)	

*Fisher’s Exact test; **Chi square test; p<0.05

In the pre-operative period, the authors verified a lower bacterial plaque index (p=0.028) and greater salivary flow for the CG (p=0.004), in comparison with the means observed in the IG. In the postoperative period of one month, the authors were already able to perceive a lower bacterial plaque index for IG (p<0**.**0001), however, in relation to salivary flow, there was no significant difference between the groups. In the postoperative period of 6M, IG recorded a lower plaque index (p<0.0001) and higher salivary flow rate (p=0.039) than CG (Table 2).

**TABLE 2:** Mean and standard deviation of plaque index and salivary flow, according to groups in pre-operative and post-operative periods of 1 and 6 months

Groups	Plaque Index			Salivary flow		
	Pre	1 month	6 months	Pre	1 month	6 months
	Mean (SD)	Mean (SD)	Mean (SD)	Mean (SD)	Mean (SD)	Mean (SD)
Control	20.07 (16.40)	50.40 (32.36)	60.55 (31.43)	1.39 (0.68)	1.05 (0.65)	1.35 (0.64)
Intervention	32.93 (28.57)	14.70 (20.22)	8.43 (15.64)	1.02 (0.51)	1.11 (0.61)	1.62 (0.70)
p	0.028	<0.0001	<0.0001	0.004	0.413	0.039

*Mann Whitney - test; p<0.05

In the comparison between CG and IG groups relative to periodontal conditions in the preoperative period, there was no significant difference for all the criteria of the community periodontal index (CPI). However, in the postoperative period of six months, there was statistical difference between the groups for gingival bleeding (p<0.0001). Better gingival conditions were observed in the bariatric patients in IG. As regards caries prevalence, according to the ICDAS criteria, in the preoperative period no statistical difference was found between the groups. Whereas, after six months, significant difference was found with regard to both changes in enamel (p=0.004), and in dentin (p=0.005). There was lower incidence of changes in IG, compared with CG (Table 3).

**TABLE 3:** Mean and standard deviation of CPI, ICDAS and DWI indices, according to groups in the pre-operative and post-operative periods of six months

Criteria	Pre-operative			Pos-operative 6 months		
	Control	Intervention	p	Control	Intervention	p
	Mean (SD)	Mean (SD)		Mean (SD)	Mean (SD)	
CPI INDEX						
Healthy	7.15 (3.07)	6.05 (3.64)	0.126	3.46 (3.85)	5.76 (4.33)	0.023
Bleeding	1.44 (2.42)	2.24 (3.11)	0.087	3.11 (3.68)	0.38 (1.31)	<0.0001
Calculus	0.35 (0.48)	0.27 (0.67)	0.107	0.63 (1.30)	0.27 (0.52)	0.060
Pocket 4-5	0.17 (0.60)	0.45 (1.50)	0.689	0.35 (1.26)	0.22 (0.93)	0.680
Pocket 6/+	0.04 (0.19)	0.02 (0.13)	0.549	0.06 (0.23)	0.15 (1.07)	0.315
ICDAS INDEX						
Without change	26.98 (4.73)	26.13 (4.15)	0.085	20.72 (11.11)	19.42 (11.99)	0.642
Change in enamel	0.87 (1.61)	0.84 (2.34)	0.660	1.61 (2.24)	0.49 (1.13)	0.004
Change in dentin	0.17 (0.50)	0.27 (0.67)	0.412	0.70 (1.42)	0.18 (0.54)	0.005
DWI INDEX						
Normal	23.37 (6.33)	20.29 (6.26)	0.003	16.57 (9.83)	14.09 (9.98)	0.158
Incipient	4.48 (4.42)	6.29 (4.84)	0.028	6.00 (5.56)	5.69 (5.71)	0.713
Moderate	0.06 (0.40)	0.27 (1.11)	0.101	0.52 (1.41)	0.22 (0.71)	0.341

*Mann-Whitney test p<0.05

When comparing CG and IG groups in the preoperative period with respect to the Dental Wear Index, the authors could observe a higher mean value for CG relative to the criterion normal (p=0,003) and for IG, relative to the criterion incipient (p=0.028). In the postoperative period of six months, there was no significant difference between groups CG and IG, for all the criteria evaluated. No severe wear was diagnosed in both groups (Table 3).

 It is worth mentioning that there was a considerable reduction in the body mass index of patients after bariatric surgery, only one patient in group IG remained in obesity III and a large portion of the patients migrated to the preobese and obesity I groups. At six months after surgery, 10% of the patients in the CG and 10.6% in IG had attained a normal BMI.

## DISCUSSION

 As far as we know, this is the first randomized clinical trial to describe the impact of an oral health promotion program developed with bariatric patients. The health promotion program implemented was shown to be effective in the prevention of the main oral health problems. Six months after the bariatric surgery, lower indices of plaque, gingival bleeding and dental caries, in addition to an increase in salivary flow, with reduction in the condition of xerostomia, were observed in the patients of IG, when compared with those in CG.

 Repeated motivational meetings and educational actions have been described in the literature, resulting in more significant improvements in the oral health knowledge and behaviors of patients, when compared to an isolated oral health guidance^12^. Linked with the individual educational activities developed during the medical review queries, the reinforcement of educational messages at regular intervals, by means of telephone contacts, described and experienced in this study by IG bariatric patients, with nutritional guidance related to oral health, as well as oral hygiene care^13^, was shown to be effective for promoting the oral health of these patients compared to those in CG. According to McGrice and Don Paul^17^, digital communication methods, such as social media, telephone consultations, and online educational programs, should be used to increase engagement with bariatric patients and to minimize barriers such as time, distance, and cost.

 In the present study, IG participants, who have undergone various approaches to guidance and motivation for bacterial plaque control, as well as supervised tooth brushing, showed a significant reduction in the plaque index. It is worth mentioning that periodic visits to the dental office were also recommended to them for plaque control. In contrast, those in CG, who received the usual care from the bariatric clinic staff, presented greater plaque accumulation in the postoperative period of one and six months, which is in accordance with the report of Hague and Baechle^9^, who emphasized the high and generalized amount of bacterial plaque observed in patients after being submitted to gastroplasty. This condition occurs, probably due to the fact that, traditionally, the oral health care has been neglected in bariatric surgeries settings.

 Regarding the periodontal conditions, in the literature there are reports of high prevalence of periodontal disease in the morbidly obese that worsens in the first six months after surgery^21^ and that there is an increase in gingival bleeding, with a peak at six months after gastroplasty^18^. Indeed, in this study, the main change relative to periodontal health observed between the groups was gingival bleeding. It could be seen that the educational actions for plaque control also exerted positive influence on the periodontal status of the IG patients. While the CG members presented a rise in gingival bleeding after the elapse of six months post-surgery, the participants in the oral health promotion program (IG), presented significant reduction in gingival bleeding (p<0.0001). It is worth noting that, unlike another survey^21^, where all teeth were evaluated, we chose to use the CPI index to optimize the evaluation time.

 It is known that bariatric patients experience great difficulty in drinking water due to stomach reduction, which tended to normalize after 12 months^4^. Inadequate water intake is of concern to both the medical field, since it is cause of dehydration, being aggravated by episodes of vomiting and diarrhea, as for dental field, considering that it contributes to dry mouth^9^. Hyposalivation is common in patients submitted to gastroplasty in the first six months after surgery^4^. Previous study have shown that there is an increase in periodontal disease and dental caries when the amount and/ or quality of saliva is reduced, as well as in masticatory disorders^27^.

 With the purpose of encouraging and assisting the water intake and improve salivary flow, IG patients received guidance relative to increasing water consumption throughout the day. The importance of taking a bottle of water with them and remembering of drinking it in small sips was constantly enhanced. This group was also advised to use chewing gum without sugar to stimulate salivation after two month of surgery. Thus, although the GI patients presented lower salivary flow than those of CG at the baseline assessment, probably due to the use of medicament with xerostomic effects, this condition was reversed in the postoperative period of six months. There was a significant increase in the mean value of salivary flow among the members of the GI when compared to that occurred among CG members. The increase in salivary flow in IG was of 58.8%.

 Previous publications have shown a tendency to increase dental caries over time after bariatric surgery^5^. The postoperative dietary recommendations for bariatric patients may be associated with this increase as they include eating food in smaller quantities, dividing food intake into 4-6 meals throughout the day chewing them slowly, and ending meals when feeling “comfortably full”^24^. The higher frequency and prolonged meal times may be related to an increase risk of dental caries, especially when taking into account that, in many instances, sweetened items are ingested by the choice of the patients^16^. Intake of dietary sugars is the most important risk factor for dental caries^29^. This eating behavior, concomitant with poor oral hygiene may result in impairment of oral health, with the development of carious lesions^9^. In this investigation, after six months, the incidence of caries was significantly lower in IG when compared with CG, affecting both enamel (p=0.004), and dentin (p=0.005). This positive finding was probably due to the reinforcing guidance adopted concerning a diet with less added sugar. Dietary advice in the dental practice is important for oral and general health preventive care^10^. Advice to eat less sugar and to reduce the frequency with which sugar foods and drinks are consumed, aiming toward a maximum of one per day, should help bring sugar intake in line with current guidelines^19^. The WHO recommends a reduced intake of free sugars for both children and adults, which should not exceed 10% of total calorie intake^29^.

 Another preventive measure that may have contributed to a lower incidence of caries in GI was the use of fluoridated varnish. A systematic review showed that two applications per year of fluoridated varnish significantly reduced caries lesions in permanent teeth^6^. In the present research, it can be assumed that the topical applications of fluoridated varnish performed in the 1^st^ month after surgery also had good results for IG by preventing caries.

 The Roux-en-Y gastric bypass is being performed in 75% of Brazilian gastroplasties^25^, justifying the fact that most of the patients studied underwent this type of surgery. The success of the technique could be verified by the weight loss recorded and the change in obesity classification among bariatric patients of the IG and CG, six months after surgery.

 Some limitations may be pointed out in the present study, such as: not all the patients returned to the clinics up to the conclusion of the research (postoperative period of six months), reducing the sample number in the final evaluation; the period of six months was relatively short for evaluating the occurrence of tooth erosion. Further researches are necessary, with longer follow-up periods, to assess the greater benefits that an oral health promotion program could have over time.

 The care protocol used in this study showed that it was possible to prevent the negative repercussions of bariatric surgery on oral health. When dentists are included in the multidisciplinary teams that care for patients submitted to bariatric surgery they can - through their relevant knowledge - contribute to the well-being of these patients.

## CONCLUSION

The oral health promotion program had a positive impact on the prevention of dental caries, periodontal disease, xerostomia and plaque accumulation in bariatric patients. These patients need to be included in an oral health promotion program with pre- and postoperative dental monitoring in order to prevent the development of oral diseases, which would contribute to improvement in their quality of life.

## References

[1] Associação Brasileira de Empresas de Pesquisa (ABEP) (2016). Critério Brasil 2015 e Atualização da Distribuição de Classes para
2016.

[2] Carvalho A da S, Rosa R dos S (2018). Cirurgias bariátricas realizadas pelo Sistema Único de Saúde em
residentes da Região Metropolitana de Porto Alegre, Rio Grande do Sul,
2010-2016*. Epidemiol e Serviços Saúde.

[3] D&apos;Aniello R, Troisi J, D&apos;Amico O, Sangermano M, Massa G, Moccaldo A (2015). Emerging Pathomechanisms Involved in Obesity. J Pediatr Gastroenterol Nutr.

[4] Dantas RO, Alves LMT, Cassiani R de A, Santos CM dos (2011). Evaluation of liquid ingestion after bariatric
surgery. Arq Gastroentero.

[5] De S Porcelli IC, Roma CC, Nunes MC, Maciel SM, Pascotto RC (2016). Effects of Bariatric Surgery on the Oral Health of
Patients. Int J Dent Oral Health.

[6] England PH (2017). Delivering better oral health: an evidence-based toolkit for
prevention.

[7] Flink H, Bergdahl M, Tegelberg A, Rosenblad A, Lagerlöf F (2008). Prevalence of hyposalivation in relation to general health, body
mass index and remaining teeth in different age groups of
adults. Community Dent Oral Epidemiol.

[8] Garcia RI, Kleinman D, Holt K, Battrell A, Casamassimo P, Grover J (2017). Healthy Futures Engaging the oral health community in childhood
obesity prevention - Conference summary and recommendations. J Public Health Dent.

[9] Hague AL, Baechle M (2008). Advanced caries in a patient with a history of bariatric
surgery. Am Dent Hyg Assoc.

[10] Harrison R, Benton T, Everson-Stewart S, Weinstein P (2007). Effect of motivational interviewing on rates of early childhood
caries: a randomized trial. Pediatr Dent.

[11] Ismail AI, Sohn W, Tellez M, Amaya A, Sen A, Hasson H (2007). The International Caries Detection and Assessment System (ICDAS)
an integrated system for measuring dental caries. Community Dent Oral Epidemiol.

[12] Kay EJ, Vascott D, Hocking A, Nield H (2016). Motivational interviewing in general dental practice A review of
the evidence. BDJ.

[13] Khan SY, Holt K, Tinanoff N (2017). Nutrition Education for Oral Health Professionals A Must, Yet
Still Neglected. J Dent Educ.

[14] Kolotkin RL, Kim J, Davidson LE, Crosby RD, Hunt SC, Adams TD (2018). 12-year trajectory of health-related quality of life in gastric
bypass patients versus comparison groups. Surg Obes Relat Dis.

[15] Mancini MC (2014). Bariatric surgery - An update for the
endocrinologist. Arq Bras Endocrinol Metabol.

[16] Marsicano JA, Grec PG de M, Belarmino LB, Ceneviva R, Peres SH de CS (2011). Interfaces between bariatric surgery and oral health: a
longitudinal survey. Acta Cir Bras.

[17] McGrice M, Don Paul K (2015). Interventions to improve long-term weight loss in patients
following bariatric surgery challenges and solutions. Diabetes Metab Syndr Obes.

[18] Moura-Grec PG, Yamashita JM, Marsicano JA, Ceneviva R, De Souza Leite CV, De Brito GB (2014). Impact of bariatric surgery on oral health conditions: 6-months
cohort study. Int Dent J.

[19] Moynihan P (2016). Sugars and Dental Caries Evidence for Setting a Recommended
Threshold for Intake. Adv Nutr.

[20] O&apos;Leary TJ, Drake RB, Naylor JE (1972). The Plaque Control Record. J Periodontol.

[21] Pataro AL, Costa FO, Cortelli SC, Cortelli JR, Dupim Souza AC, Nogueira Guimarães Abreu MH (2012). Influence of Obesity and Bariatric Surgery on the Periodontal
Condition. J Periodontol.

[22] Sales-Peres SHC, Maia-Júnior AF*, Bastos JRM S-PA (2006). PO050 Estudo de prevalência e de severidade de facetas de
desgaste dentário, em adultos jovens. Braz Oral Res.

[23] Sheiham A (2005). Oral health, general health and quality of life. Bull World Health Organ.

[24] Sherf Dagan S, Goldenshluger A, Globus I, Schweiger C, Kessler Y, Kowen Sandbank G (2017). Nutritional Recommendations for Adult Bariatric Surgery Patients
Clinical Practice. Adv Nutr.

[25] Sociedade Brasileira de Cirurgia Bariátrica e Metabólica (2018). Número de cirurgias bariátricas no Brasil aumenta 46,7%.

[26] Sturm R, Hattori A (2013). Morbid obesity rates continue to rise rapidly in the United
States. Int J Obes.

[27] Tremblay M, Brisson D, Gaudet D (2012). Association between salivary pH and metabolic syndrome in women a
cross-sectional study. BMC Oral Health.

[28] World Health Organization (2013). Oral health surveys: basic methods - 5th edition.

[29] World Health Organization (2015). Nutrition for Health and Development. Guideline: Sugars intake for
adults and children.

[30] World Health Organization (2000). Obesity: preventing and managing the global epidemic: report of a WHO
consultation.

